# 2592. The Incidence Rate Of Pneumococcal Pneumonia In Two European Aging Adults Population Using Real-World Data

**DOI:** 10.1093/ofid/ofad500.2207

**Published:** 2023-11-27

**Authors:** Mónica López-lacort, Marzyeh Amini, Hanne-Dorthe Emborg, Jens Nielsen, Palle Valentiner-Branth, Brita Askeland Winje, Anneke Steens, Scott A McDonald, Kelly D Johnson, Miloje Savic, Javier Díez-Domingo, Alejandro Orrico-Sánchez

**Affiliations:** Vaccine Research department of Fisabio-Public Health, Valencia, Comunidad Valenciana, Spain; P95 Epidemiology and Pharmacovigilance, Leuven, Brussels Hoofdstedelijk Gewest, Belgium; Department of Infectious Disease Epidemiology and Prevention, Copenhage, Nordjylland, Denmark; Department of Infectious Disease Epidemiology and Prevention, Copenhage, Nordjylland, Denmark; Department of Infectious Disease Epidemiology and Prevention, Copenhage, Nordjylland, Denmark; Faculty of Health Sciences, Oslo, Oslo, Norway; Center for Infectious Disease Control, National Institute for Public Health and the Environment (RIVM), Amsterdam, Noord-Brabant, Netherlands; Center for Infectious Disease Control, National Institute for Public Health and the Environment (RIVM), Amsterdam, Noord-Brabant, Netherlands; Center for Observational and Real World Evidence (CORE) Vaccines, Merck, phyladelphia, Pennsylvania; Epidemiology, GSK, Brussels, Brussels Hoofdstedelijk Gewest, Belgium; FISABIO, Valencia, Comunidad Valenciana, Spain; FISABIO-Public Health, Valencia, Comunidad Valenciana, Spain

## Abstract

**Background:**

Pneumococcal pneumonia (PP) is a major infectious cause of morbidity and mortality worldwide. It is occurring predominantly in the elderly and those with co-morbidities. To guide policymakers about prevention strategies it is essential to understand the disease occurrence in the population. In the context of the Vaccines and InfecTious Diseases in the Aging PopuLation (VITAL) project, this study aimed to assess PP incidence rates in aging adults (50+ years) in two European countries, Spain and Denmark, using real-world data.

**Methods:**

We conducted a retrospective analysis within the electronic healthcare record (EHR) of the Valencia Health System Integrated Databases of Spain and Statens Serum Institute Database of Denmark. We identified hospitalized invasive PP (IPP) and non-invasive PP (NIPP) cases occurring from January 1, 2010 through December 31, 2018, using a combination of microbiological testing and clinical (ICD9/10 diagnoses for disease manifestations) data recorded within the EHR. Overall incidence per 100,000 person-years (PYs) was estimated and stratified by age group, sex, underlying condition, and calendar year.

**Results:**

A study population of 2,283,344 and 2,782,303 aging adults were included from Valencia and Denmark, respectively. The overall incidence rate of IPP for all years collectively was higher in the Danish than in the Valencia population (18.3 vs. 9.8/100,000 PYs), while the incidence rate of NIPP in the Valencia population was substantially higher (48.2 vs. 7.2/100,000 PYs). The incidence of IPP increased along with age, ranging from 3.9/100,000 PYs in 50-55 years-old to 23.0 in ≥80 years-olds in Valencia, and 5.9/100,000 PYs in 50-55 years-old to 47.3 in ≥80 years-old in Denmark. The incidence rate of NIPP showed the same age-group trend. In both countries, the incidence rate of (N)IPP differed over the years and in both sex but was higher in patients with underlying conditions.

**Conclusion:**

The incidence rates of (N)IPP fluctuated over the years and increased with age in both countries. However, substantial between-country variation was observed. The differences in incidence rates in two geographically distinct countries may be in large parts attributable to bias due to testing rates and coding practice, and for IPP also on pre-hospital antibiotic use.

**Disclosures:**

**Mónica López-lacort, n/a**, GSK: Grant/Research Support|Merck: Grant/Research Support|Merck: Honoraria|Sanofi: Grant/Research Support **Kelly, D Johnson, n/a**, Merck: Stocks/Bonds **Miloje Savic, PhD**, GSK Biologicals: Stocks/Bonds **Alejandro Orrico-Sánchez**, GSK: Grant/Research Support|Merck: Advisor/Consultant|Merck: Board Member|Merck: Grant/Research Support|Merck: Honoraria|Pfizer: Grant/Research Support|Sanofi: Board Member|Sanofi: Grant/Research Support|Sanofi: Honoraria

